# Evaluation of the apposition in unruptured aneurysms treated with flow diverters by optical coherence tomography: Preliminary clinical experience

**DOI:** 10.3389/fneur.2022.1029699

**Published:** 2022-10-24

**Authors:** Jing Li, Wentao Gong, Dongdong Li, Wenpeng Song, Feng Fan, Yongjie Yuan, Youxiang Li, Sheng Guan

**Affiliations:** ^1^Department of Neurointervention Radiology, The First Affiliated Hospital of Zhengzhou University, Zhengzhou, China; ^2^Department of Interventional Neuroradiology, Beijing Tiantan Hospital, Capital Medical University, Beijing, China

**Keywords:** apposition, flow diverter, optical coherence tomography, aneurysm, stent

## Abstract

**Background:**

The risk of perioperative stroke and the rate of occlusion of long-term aneurysms in the treatment of unruptured aneurysms with flow diverters (FDs) are affected by stent apposition. Optical coherence tomography (OCT) may be an optional technique in evaluating apposition.

**Purpose:**

To explore the feasibility of the OCT imaging technique in evaluating stent apposition in the clinical application of the FD for unruptured aneurysms.

**Methods:**

OCT and Vaso CT were used in patients with indications for surgery to treat unruptured aneurysms with the FDs, to evaluate the apposition of the FDs after fully released, and to analyze OCT images for FDs apposition and compare with corresponding Vaso CT images.

**Results:**

A total of four patients were enrolled, and OCT found malapposition after FDs placement in all four patients, and the maximum gap between the stent and vascular wall ranged from 0.68 to 1.95 mm and the length of malapposition ranged from 1.80 to 7.40 mm. However, Vaso CT found malapposition only in two of the four patients and missed malapposition near aneurysm in all three patients treated by the FD combined with coiling and could not accurately evaluate the maximum gap and the length of the malapposition.

**Conclusion:**

The optical coherence tomography technique is a possible approach to evaluate apposition after the treatment of unruptured aneurysms by the FDs.

## Introduction

The efficacy and safety of the FDs for the treatment of unruptured intracranial aneurysms are being confirmed by a growing number of clinical studies (1). Delayed endothelial growth due to inadequate release and malapposition of the FDs is one of the important factors reducing the occlusion rate of aneurysms (2–4), as blood continues to flow to the aneurysm sac through the gap between the malapposition and the vascular wall, preventing the aneurysm from occlusion (5). In addition, malapposition can lead to complications such as stenosis, thrombosis, and distal thromboembolism (6). Therefore, whether the FDs have sufficiently adhered to the vascular wall determines the perioperative and long-term safety and effectiveness of the treatment.

Optical coherence tomography is an increasingly used technique to evaluate the apposition after coronary stent implantation because of its high resolution (micron level) and no metal interference in imaging. At present, most of the studies using OCT to evaluate the apposition of the FDs are mostly limited to animal models, whereas studies on clinical applications are rarely reported. In this research, we report four clinical cases, intending to provide more clinical experience in the use of OCT to evaluate FDs apposition in comparison with Vaso CT.

## Methods

### Patients screening

The Ethics Committee of the First Affiliated Hospital of Zhengzhou University approved the study. A total of four adult patients were included in the study from May 2021 to March 2022. DSA imaging features of the study population (*n* = 4) were collected and evaluated by two experienced neurointerventors, all of whom met the surgical indications for the treatment of unruptured intracranial aneurysms with the FDs. All patients or their immediate relatives signed informed consent to the study.

### Study design

All patients completed imaging collection such as DSA, Vaso CT, and OCT immediately after the FD fully and smoothly inserted, and the degree of stent apposition was evaluated based on the above various imaging techniques, of which communicating malapposition (CM) was defined as poor apposition (distance between FD and vessel wall >200 μm) (7) that continues to the aneurysm neck. According to clinical experience and the results of OCT imaging evaluation and taking into consideration the safety of the procedure, the surgeon decides whether to continue or end the operation. The patient's baseline features (age and sex), aneurysm features (location, type, neck width, maximum height, maximum diameter, proximal and distal reference diameter of the parent artery), surgical details (type, size, and number of FDs used, and whether coil embolization is applied), and OCT image features during the perioperative period were collected for further comparative analysis (Table 1).

**Table 1:** Clinical details of the patients.

	**Case 1**	**Case 2**	**Case 3**	**Case 4**
Age (year)	35	40	47	64
Sex	Male	Female	Male	Female
Aneurysm location	RICA C1	RICA C1	LVA V4	LVA V4
Aneurysm type	Saccular	Saccular	Saccular	Fusiform
Aneurysm neck (mm)	8.79	11.38 (Upper)	5.83	11.66
		8.61 (Lower)		
Maximum height (mm)	10.21	12.86 (Upper)	4.96	9.09
		7.66 (Lower)		
Maximal diameter (mm)	19.49	14.81 (Upper)	8.97	11.66
		12.21 (Lower)		
Proximal artery diameter (mm)	5.18	4.45	3.32	4.35
Distal artery diameter (mm)	5.67	4.62	3.36	4.06
Type of FD	Repath	Repath	Tubridge	Tubridge
Number of FDs	1	1	1	2
Size of FD (mm)	6.0*45	5.0*60	4.0*25	5.0*25
				5.0*35
Additional coiling	Yes	Yes	Yes	No
OCT method	Non-occlusive	Non-occlusive	Non-occlusive	Non-occlusive

RICA, right internal carotid artery; LVA, left vertebral artery; FD, flow diverter; OCT, optical coherence tomography.

### Endovascular procedure and acquisition of OCT images

The procedures of FD placement are given in the following paragraphs. All four patients were under general anesthesia, systemic heparinization, and femoral artery approach. For case 1 and case 2, an 8F short sheath (Terumo, Japan) was inserted and a 6F NeuroMax catheter (Genesis MedTech, China) was delivered to the beginning of the internal carotid artery. In the path diagram, an Echelon-10 microcatheter (Medtronic, USA) was placed in the aneurysm guided by a 0.014-in Synchro-2 microwire (Stryker, USA) and an XT-27 microcatheter (Stryker, USA) was placed in the C3 segment of the internal carotid artery. Delivered by the XT-27 microcatheter, Repath FD was precisely positioned and half-released, the aneurysm was sparsely packed along the Echelon-10 microcatheter, and the FD was fully released after the coiling was finished. For intracranial vertebral artery aneurysms, a 6F short sheath (Terumo, Japan) was inserted and a 6F long sheath (Cook, USA) was replaced. The long sheath was placed at the proximal of the left vertebral artery opening, and a 5F Catalyst intermediate catheter (Stryker, USA) was delivered to the left vertebral artery V2 segment. A T-Track microcatheter was placed at the proximal basilar artery guided by a 0.014-in Synchro-2 microwire. For case 3, the Tubridge FD (MicroPort Medical, China) was delivered along the T-Track (MicroPort Medical, China) microcatheter, then precisely positioned, and half-released, introducing the Echelon-10 microcatheter into the aneurysm and filling the coils. Then, the FD was fully released. For case 4, the Tubridge FD was directly delivered, precisely positioned, and completely released without coiling in advance, and then, Vaso CT suggests that although the proximal section of the stent covered the aneurysm neck, the proximal opening was too close to the proximal end of the aneurysm neck. The second Tubridge FD was released using the above method.

An OCT image acquisition device is an Ilumien Optis System (St. Jude Medical, USA), which uses a non-occlusive method. After the FD was fully released, the Dragonfly Duo OCT imaging catheter was delivered and positioned at the distal end of the region of interest. The drawdown speed was set to 18 mm/s, the drawdown distance to 54 mm, and the imaging speed to 180 fps. The OCT images were acquired using automatic high-pressure syringe pumps (Mark V ProVis, Germany) for simultaneous injection of undiluted isotonic contrast (GE Healthcare, Ireland), setting the flow speed (carotid artery 4 ml/s, vertebral artery 3 ml/s), flow volume (14 ml), and maximum injection pressure (300 psi). The contrast was injected manually into the guide catheter to remove blood from the duct cavity and injected again under DSA to determine the clearance effect. After the automatic high-pressure syringe pump injected the contrast agent at the beginning of the scan (the total amount of contrast agent was quickly injected through the guide catheter) and after the system recognized the image clarity, it automatically retracted and acquired the images. The OCT images were used in the Ilumien Optis System software (St. Jude Medical, USA) and RadiAnt DICOM Viewer (Medixant, Poland) for measurement analysis.

### Acquisition of Vaso CT images

We obtained Vaso CT images with Philips Azurion 7M20 Biplane DSA (Philips Medical Systems, The Netherlands). We selected the Vaso CT option in the operation module, placed the area of interest in the center of the scan, adjusted the rate of hyperbaric syringe injection (carotid aneurysm 4 ml/s, spinal aneurysm 3 ml/s), and injected a 50% non-ionic contrast medium iohexol 370 mg I/ml diluted by isotonic saline into the vascular lumen (carotid aneurysm 80 ml/s, vertebral aneurysm 60 ml/s), with the continuous rotation of the DSA machine from +120° to −120° in 20 s (carotid aneurysm delay of 0 s, vertebral aneurysm delay of 1 s). Acquired data were scanned and transmitted to a 3D workstation for processing. The image rotation, the appropriate angle selection, and the application of maximum density projection were applied to observe the image clarity and the degree of apposition of FD.

## Results

A total of four patients with five aneurysms were included in this study. There were three cases of anterior circulation aneurysms and two cases of posterior circulation aneurysms, among which anterior circulation aneurysms were located in the external cranial segment of the internal carotid artery and posterior circulation aneurysms were located in the intracranial segment of the vertebral artery. The average maximum diameter of the five aneurysms was 13.43 mm. Four out of five cases belong to the large aneurysm type (maximum diameter 11–25 mm) and one case of the medium aneurysm type (maximum diameter of 5–10 mm). Among the four patients, three patients were placed with the FD and loosely packed with coils and the other patient was placed in the second FD, considering that the proximal portion of the stent was too close to the aneurysm sac after being inserted. There was no surgery-related ischemia or bleeding complications in the four patients. All patients successfully completed Vaso CT and OCT image acquisition immediately after FD placement.

### Case 1

**Figure 1:**
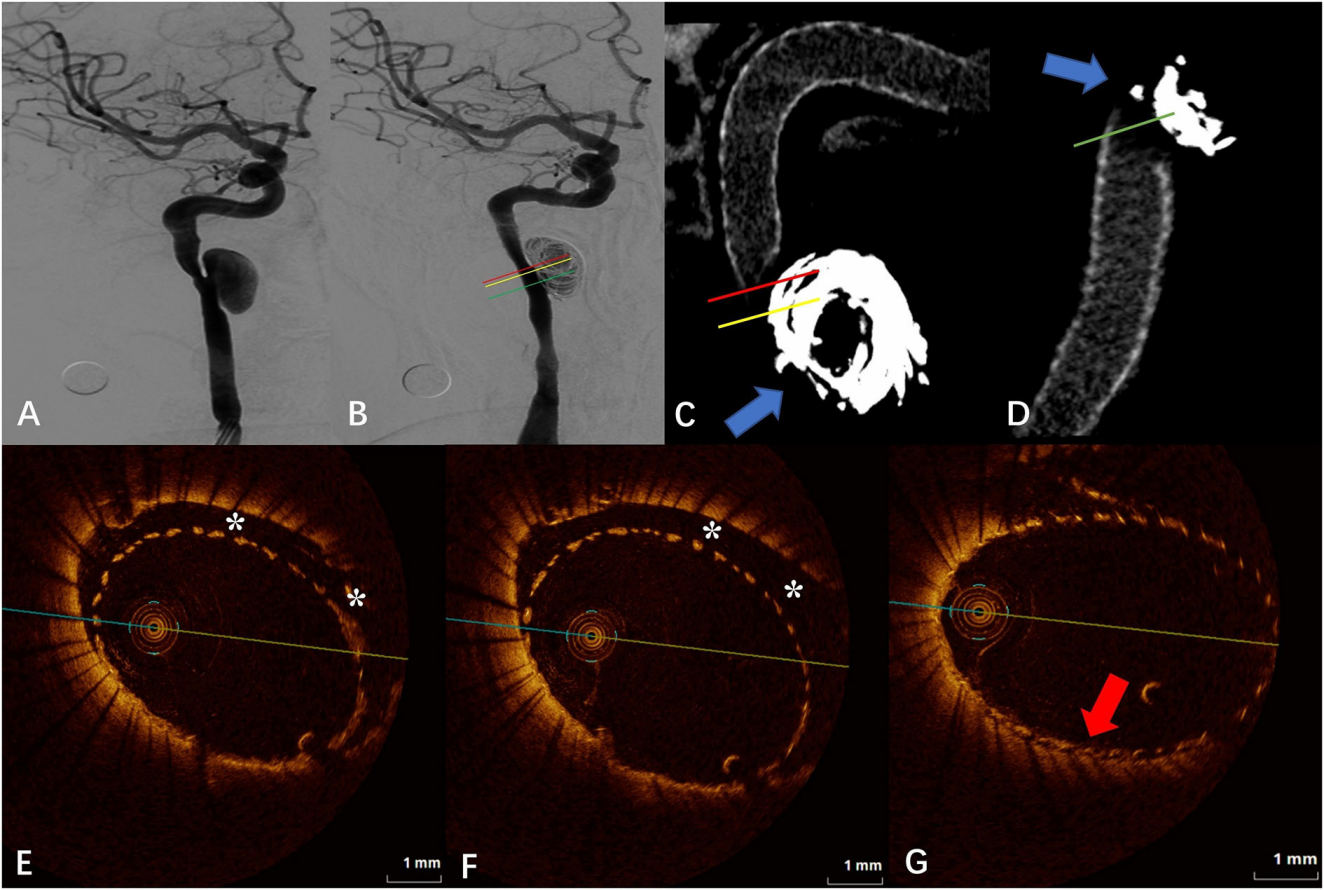
**(A)** Digital subtraction angiography (DSA) before the flow-diverter placement. **(B)** Digital subtraction angiography (DSA) just after the flow-diverter placement. **(C,D)** Vaso CT images. Distal and proximal parent artery with good apposition of the FD to the wall. Artifacts near the aneurysm neck that disturb the evaluation of apposition by Vaso CT (blue arrows). **(E,F)** OCT images. The corresponding red **(E)** and yellow **(F)** line in **(B,C)**. Eccentric communication malapposition near the aneurysm (*). **(G)** OCT images. The corresponding green line in **(B,D)**. Good FD apposition at the aneurysm neck (red arrow).

The DSA images before and after FD placement are shown in Figures 1A,B, respectively. The Vaso CT images (Figures 1C,D) showed that the distal and proximal ends of the stent were well adhered to the vascular wall, and due to the influence of the metal artifacts of the coils, the stent structures that contacted with the coils were poorly observed (blue arrows in Figures 1C,D). The OCT image showed a long-segment CM of the stent at the neck of the aneurysm, which is 6.20 mm long, and the maximum distance between the stent and the vascular wall is 0.79 mm. The degree of FD apposition in OCT is shown in Figures 1E–G.

### Case 2

**Figure 2:**
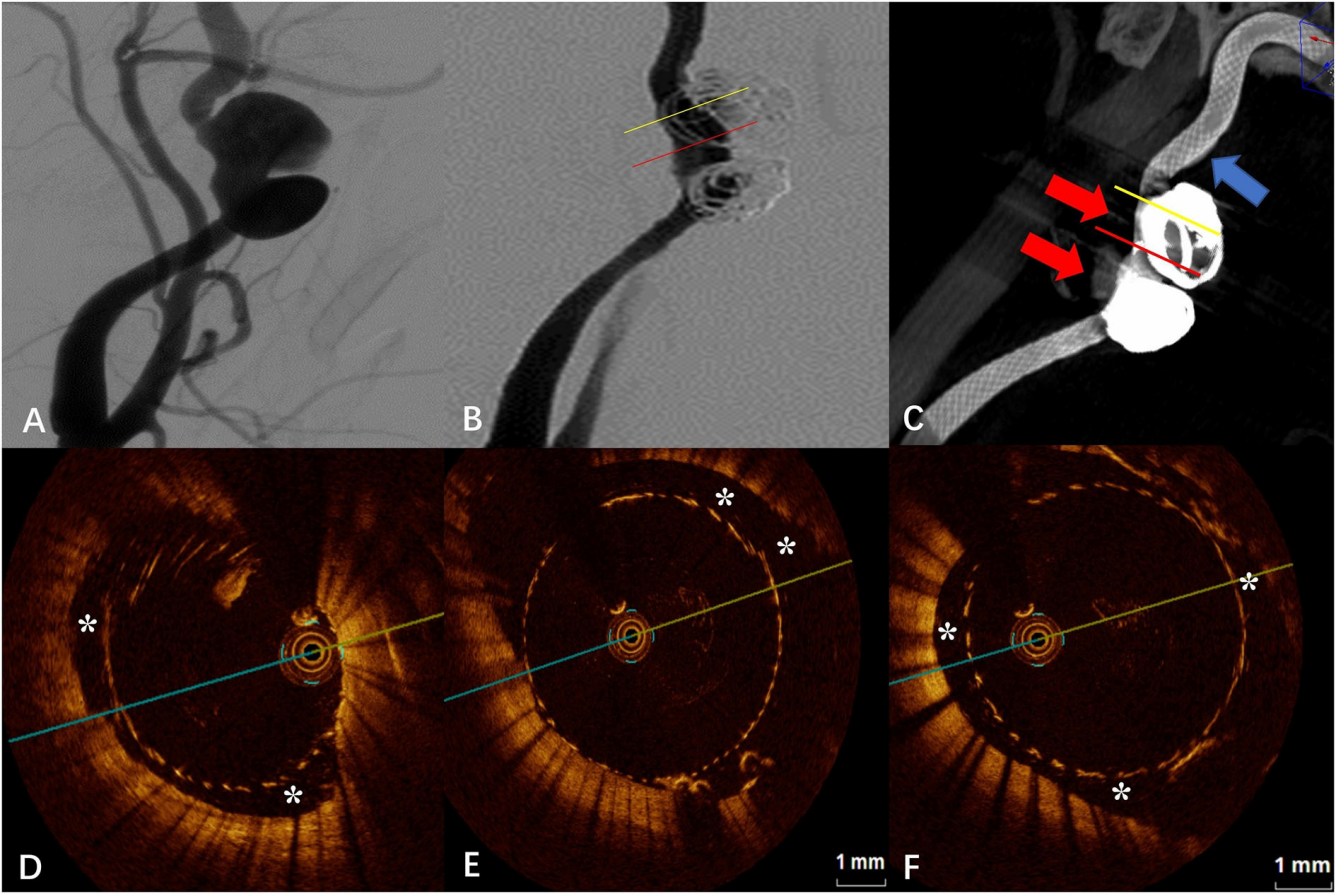
**(A)** Digital subtraction angiography (DSA) before the flow-diverter placement. **(B)** Digital subtraction angiography (DSA) just after the flow-diverter placement. **(C)** Vaso CT images. Localized malapposition in distal parent artery (blue arrow) and proximal parent artery with good apposition of the FD to the wall. Artifacts near the aneurysm neck that disturb the evaluation of apposition by Vaso CT (red arrows). **(D)** OCT image. Corresponding to localized malapposition [blue arrow in **(C)**] confirmed by Vaso CT (*). **(E)** OCT image. The corresponding yellow line in **(B,C)**. Eccentric communication malapposition near the aneurysm neck (*). **(F)** OCT image. The corresponding red line in **(B,C)**. Circumferential communication malapposition at the aneurysm (*).

The DSA images before and after FD placement are shown in Figures 2A,B, respectively. The Vaso CT images showed localized malapposition of the FD at the distal end of the neck of the upper aneurysm (blue arrow in Figure 2C), and the rest were well adhered to the vascular wall. The junction between the stent and the coils was affected by metal artifacts and cannot be evaluated (red arrows in Figure 2C). The OCT images showed the localized malapposition (“^*^” in Figure 2D) in the area corresponding to the blue arrow in Figure 2C. Eccentric CM can be seen near the aneurysm neck and distal portion of the stent (“^*^” in Figure 2E), with a length of about 2.40 mm and a maximum gap of 0.93 mm between the stent and the vascular wall. The upper aneurysm neck can be seen with localized circular malapposition of the stent (“^*^” in Figure 2F), with a length of about 1.80 mm and a maximum gap between the stent and the vascular wall of 0.62 mm. The CM of the neck of the lower aneurysm is mainly limited to the posterior wall and lateral wall of the parent artery (the aneurysm position is anterior), with a length of about 5.60 mm and a maximum gap between the vascular walls of about 1.95 mm. The proximal portion of the stent can also be seen with malapposition, circular at some point, which does not extend to the distal end to the neck of the aneurysm, with a length of about 5.60 mm and a maximum gap between the stent and the vascular wall of about 0.94 mm.

### Case 3

**Figure 3:**
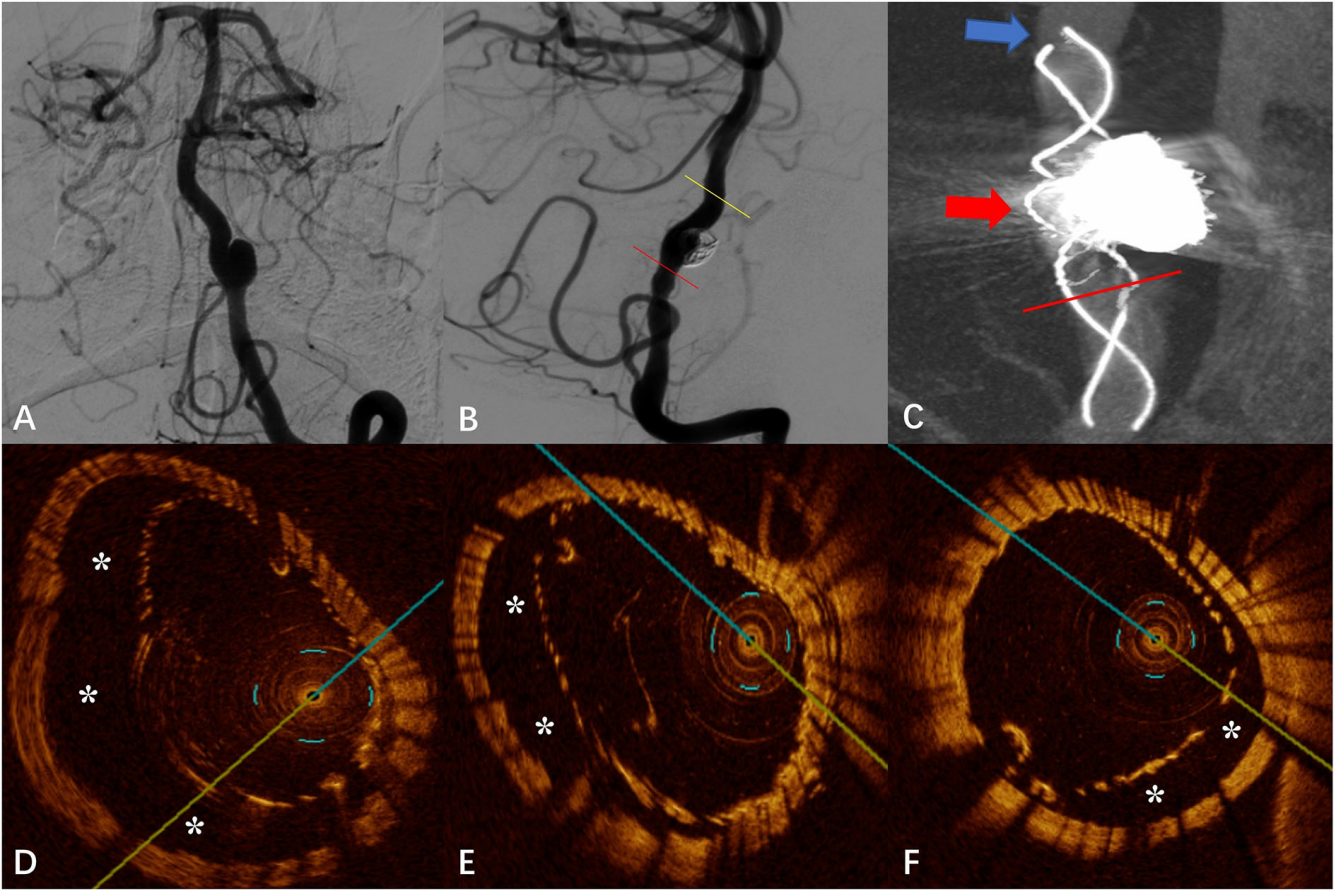
**(A)** Digital subtraction angiography (DSA) before the flow-diverter placement. **(B)** Digital subtraction angiography (DSA) just after the flow-diverter placement. **(C)** Vaso CT image. Malapposition at the head of the FD (shown by blue arrow) and proximal parent artery with good apposition to the wall. Artifacts near the aneurysm neck that disturb the evaluation of apposition by Vaso CT (shown by red arrow). **(D)** OCT image. The corresponding blue arrow in **(C)**. Malapposition at the head of the FD confirmed by Vaso CT before the “massage” of the J-shaped guidewire (*). **(E)** OCT image. The corresponding yellow line in **(B)**. Malapposition at the head of the FD confirmed by Vaso CT after the “massage” of the J-shaped guidewire (*). **(F)** OCT image. The corresponding red line in **(B,C)**. Localized non-communication malapposition at the proximal of the aneurysm neck.

The DSA images before and after FD placement are shown in Figures 3A,B, respectively. The Vaso CT image showed suspected malapposition at the distal end of the stent (blue arrow in Figure 3C) and good apposition at the proximal artery. The junction between the stent and the coils was affected by metal artifacts and cannot be evaluated (red arrow in Figure 3C). OCT imaging also showed the malapposition at the same location with a length of 7.40 mm and a maximum gap of 0.94 mm (“^*^” in Figure 3D). We applied the “J” guidewire to thoroughly massage the head end of the FD several times to promote FD release. Further OCT imaging revealed improved malapposition (“^*^” in Figure 3E) compared with the previous one with a length of 4.40 mm and a maximum gap of 0.87 mm. The local proximal part of the FD is malapposition, with a length of 2.60 mm and a maximum gap of 0.68 mm, which does not extend to the aneurysm neck (“*” in Figure 3F).

### Case 4

**Figure 4:**
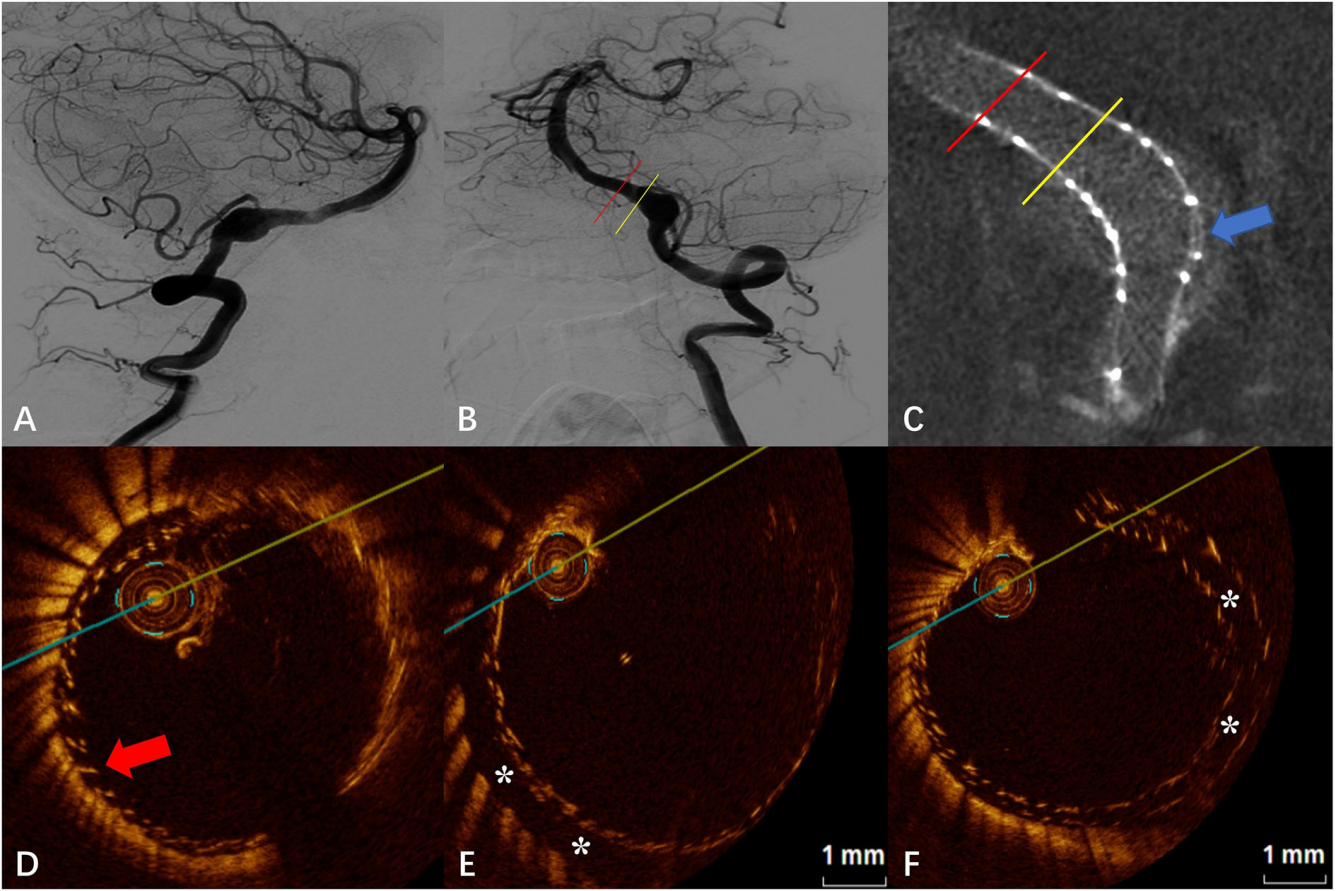
**(A)** Digital subtraction angiography (DSA) before the flow-diverter placement. **(B)** Digital subtraction angiography (DSA) just after the flow-diverter placement. **(C)** Vaso CT image. Distal and proximal parent artery with good apposition of the FD to the wall. Malapposition between the stents at the aneurysm neck (blue arrow). **(D)** OCT image. The corresponding red line in **(B,C)**. Good apposition between stents and between the stent and the wall at the distal of the aneurysm neck (red arrow). **(E)** OCT image. The corresponding yellow line in **(B,C)**. Eccentric communication malapposition at the distal of the aneurysm neck (*). **(F)** OCT image. The corresponding blue arrow in **(C)**. Malapposition between the stents at the aneurysm neck (*).

The DSA images before and after FD placement are shown in Figures 4A,B, respectively. After placement of the first FD, Vaso CT indicated that the proximal portion of the FD covered the aneurysm neck but the proximal opening of the FD was too close to the aneurysm neck (a distance of 0.80 mm, later confirmed by OCT), and the second FD was placed to adequately cover the aneurysm neck and the proximal parent artery. Subsequent Vaso CT images showed good FD apposition at both the distal and proximal parent artery, with limited inter-stent malapposition at the aneurysm neck (blue arrow in Figure 4C). The OCT images showed that the distal part of the FD was well attached to the vascular wall, and the double stents were well attached to each other with the maximum gap between the stents being <0.20 mm (red arrow in Figure 4D). Eccentric CM was seen in the lateral wall of the parent artery at the aneurysm neck (“^*^” in Figure 4E), with a length of 3.00 mm and a maximum gap of 0.69 mm. Although the outer FD was well attached to the wall in the middle and distal part of the aneurysm neck and in the distal parent artery, the malapposition between the stents was observed, especially at the aneurysm neck (“^*^” in Figure 4F), with a maximum gap of about 0.57 mm.

## Discussion

In the four cases of intracranial aneurysms treated with the FD in our study, all of them were successfully evaluated for apposition by OCT after FD placement. The results showed that the OCT technique can clearly evaluate the apposition of the FD. To our knowledge, this is the first report of clinical experience with OCT assessment of FD apposition in a series of patients with unruptured intracranial aneurysms treated with the FDs.

The application of FDs for the treatment of intracranial aneurysms, especially wide-necked, giant aneurysms, has been gradually accepted. Compared with traditional interventional modalities, the FD has the advantages of a low technical threshold, a relatively short procedure time, and a high long-term complete occlusion rate of aneurysms. The FD is placed in the parent artery covering the aneurysm neck, which reduces the blood flow in the aneurysm sac and results in thrombosis. Endothelial cells covering the stent reconstruct the parent artery and finally obtain the aneurysm occlusion (2). In this process, delayed endothelial growth on the stent surface due to malapposition after the release of the FDs is one of the important factors affecting the occlusion rate of the aneurysm (2, 3, 8). While DSA and Vaso CT are currently the most commonly used methods to assess the degree of FD apposition in the immediate postoperative period, the development of the OCT technique offers a new possibility in terms of assessment tools.

Optical coherence tomography is an emerging endoluminal imaging technique for coronary artery endovascular treatment, which not only enables imaging of the fine structure of the vessel wall but also evaluates the relationship between the stent and the vessel wall. Current studies related to the application of OCT to evaluate the FDs for the treatment of intracranial aneurysms are mostly limited to animal models. In a study with the New Zealand white rabbit model (subclavian artery), King et al. (8) found that OCT can clearly show the structure of the FD and its relationship with the vascular wall. The limitation of the animal model is that the artery path and aneurysm structure are simpler in the animal model compared with the human, while the intracranial artery in humans is more tortuous and the aneurysm morphology is more complex. After FD placement, the relationship between the stent, the parent artery, and the aneurysm is more complicated than in animal models. After FD placement, the relationship between the stent, the parent artery, and the aneurysm is more complex than in animal models, which raises additional requirements and challenges for both the performance of the OCT catheter into position and the imaging capabilities post-positioned (9). Neira et al. (5) reported a clinical case in which OCT was applied to evaluate the short- and long-term results of the FD for aneurysms of the ophthalmic segment of the internal carotid artery. After the OCT catheter was successfully positioned, FD apposition and long-term endothelialization of the stent were accurately demonstrated. Mario et al. (9) also displayed a case of successful application of OCT imaging and analysis of stent apposition of the aneurysm in post-communication artery after FD treatment.

Optical coherence tomography has more advantages than DSA and Vaso CT in evaluating post-release apposition of flow-guided devices at both qualitative and quantitative levels (10), especially for CM. CM refers to malapposition in the parent artery near the aneurysm that continues into the aneurysm neck, where the endothelium continues to fail to grow and blood continues to flow through the gap between the poorly apposed stent and the vessel wall into the aneurysm sac, creating a larger neck area and thus delaying or preventing aneurysm occlusion. One study showed that the application of OCT to assess CM predicted the rate of aneurysm occlusion at 30 days after FD placement (8). In our study, the FD combined with the coil embolization technique used in both case 1 and case 2 was to treat large internal carotid artery dissecting aneurysms. We can observe from the Vaso CT images that artifacts are evident near the aneurysm neck due to the presence of the coils, and it is not possible to make an accurate evaluation of FD apposition. The projection characteristics of conventional DSA cannot always detect eccentric CMs, and furthermore, some CMs are beyond the spatial resolution capability of DSA (8). From Figures 1–4, it is observed that OCT clearly detected and localized the CM after FD release and was able to apply post-processing software to calculate the length of the CM and the maximum gap between the stent and the vascular wall. To our knowledge, the concept of the length of CM was first proposed by us, and evaluating and defining the severity of CM and classifying it by quantitative means may facilitate to analyze and establish an effective model for predicting aneurysm occlusion by OCT method for FD treatment of intracranial aneurysms, and also deepen the understanding of aneurysm occlusion and parent artery remodeling (10). A recent study suggests that early aneurysm occlusion can be accurately predicted by calculating the area and volume of malapposition in the OCT images (7). However, most of the available OCT devices do not allow immediate intraoperative calculation of area or volume indexes, which is not favorable for rapid intraoperative evaluation of the relationship between the degree of malapposition and prognosis and determination of whether malapposition needs to be improved by additional surgical strategies (balloon dilation, remedial stent placement, etc.).

The evaluation of post-release apposition of the FD by OCT is also useful to optimize the surgical procedure. In case 3, after FD release, imaging by Vaso CT as well as OCT revealed malapposition at the head of the FD, suggesting that the head was not adequately released. Due to the small number of visualizing filaments of the Tubridge stent, the Vaso CT images are limited in assessing the extent of malapposition. The OCT technique provides a more accurate view of the stent structure as well as an accurate measurement of 7.40 mm of the length of CM and 0.94 mm of the maximum gap between the stent and the vascular wall. Moreover, the CM length was shortened to 4.40 mm and the maximum gap was reduced to 0.87 mm when OCT was reviewed after applying techniques to facilitate further FD release. A more objective and quantitative assessment of OCT can reflect the improvement in malapposition after the application of technical operation.

Furthermore, for the first time, this study reports the application of OCT to evaluate the apposition characteristics of the stent to vascular walls and inter-stents in two Tubridge (11) stents overlapped for intracranial aneurysms. In case 4, although the two stents were identical in size and the parent artery was relatively straight throughout the release, the overlapping segments of the stents did not fit tightly all the way. The OCT image demonstrated the two stents fit tightly with the maximum gap between the stents <0.20 mm at the distal and proximal part of the overlapping segment. However, a gap between the two stents was found at the aneurysm neck, with a maximum of approximately 0.57 mm. Few studies have been available on the evaluation of apposition after overlapping two or more FDs. Neira et al. (5) reported a case of recurrence after LVIS-assisted coil embolization of an aneurysm treated with the FD further and obtained a good prognosis after 1 year, in which the FD was evaluated by OCT for good FD apposition. However, stent overlap may bring a series of problems, including in-stent thrombosis, in-stent restenosis, adverse mechanical reactions (stent fracture), and even hemodynamic abnormalities, and the above-mentioned adverse events have been confirmed in the field of coronary stents (12, 13). Although in terms of endothelialization, it has been suggested that stent overlap may not affect the degree and type of stent endothelialization (14). But in the field of coronary stents, stent overlap does not seem to exist up to a 0.57-mm stent gap. Although no adverse events occurred in the perioperative period in case 4, the long-term aneurysm occlusion effect of double stenting with inter-stent malapposition at the aneurysm neck remains to be confirmed by later follow-up and more case studies. Additionally, the OCT images (Figure 4E) revealed that further stent overlap may not eliminate CM after the first stent, but more clinical studies are needed to determine whether it helps to improve the long-term prognosis.

Currently, no standardized thresholds have been established for the definition of poor FD apposition. Some have defined an FD-to-wall distance of >50 μm (approximately 1.5 times strut thickness) as malapposition (7), but the relationship between this threshold and clinical outcomes is not well understood. Threshold values that are too low may have more false-positive results in identifying malapposition. More relevant studies are now from the field of cardiovascular endovascular therapy. It was found that malapposition with stent-to-wall distance <270 μm achieved complete wall apposition and complete endothelialization at 6 months, whereas malapposition with distance ≥850 μm showed delayed persistent malapposition and endothelialization in all cases at long-term follow-up (15). In the ILLUMIEN III study, stent distance to the wall ≥200 μm was an independent predictor of major adverse cardiovascular events, while distance was acceptable in the range of <200 μm (16). The European Association of Percutaneous Coronary Intervention recommends that a distance of ≥400 μm and a length of ≥1 mm or more are defined as significant stent malapposition on the OCT image after percutaneous coronary intervention (PCI) (17). As can be seen, even in the field of cardiovascular interventions, the definition of thresholds remains somewhat controversial. For FD, more clinical studies are expected to explore reasonable, clinically guided thresholds for malapposition.

Although our study confirmed the feasibility and advantages of OCT in evaluating stent apposition after the FD for unruptured intracranial aneurysms (compared with DSA and Vaso CT) and innovatively proposed an OCT-based metric for quantitative evaluation of CM that may help predict the rate of long-term aneurysm occlusion, our study has some limitations. The number of cases in this study is small, the characteristics of the included aneurysms are different, the types of stents used are diverse, and the treatment methods include single FD, the FD combined with coil embolization, and multiple FD, which are not generally representative and still need to be confirmed by clinical studies with larger samples.

## Conclusion

With the current available neurointerventional devices, imaging in the extracranial segment of the internal carotid artery and the intracranial segment of the vertebral artery can be accomplished by the OCT catheter. The OCT technique allows a clear demonstration of stent apposition after FD treatment of unruptured intracranial aneurysms and quantitative analysis, including the maximum gap of the stent from the vascular wall and the length of malapposition. Besides, the combined application of coil embolization during FD treatment did not affect the OCT evaluation, whereas Vaso CT was affected by the coil in the evaluation of stent apposition, with no quantitative measurement assessment. The use of OCT may be useful to gain further insight and explore the relationship between FD apposition and long-term aneurysm occlusion.

## Data availability statement

The original contributions presented in the study are included in the article/supplementary material, further inquiries can be directed to the corresponding authors.

## Ethics statement

The studies involving human participants were reviewed and approved by the Ethics Committee of The First Affiliated Hospital of Zhengzhou University. The patients/participants provided their written informed consent to participate in this study. Written informed consent was obtained from the individual(s) for the publication of any potentially identifiable images or data included in this article.

## Author contributions

JL and WG drafted the manuscript, was involved in data organization and selection, data interpretation, and was a guarantor. DL and WS collected and classified the references and proofread the manuscript. FF was involved in data interpretation and proofreading of the manuscript. YY proofread the manuscript. YL and SG developed and designed the overall methodology, established the overall research goals and purposes, proofread the manuscript, and were guarantors. All authors contributed to the article and approved the submitted version.

## Funding

This research was funded by the Henan Provincial Science and Technology Research Project (application of OCT system to study the effect of flow diverters on blood vessel wall; number 222102310195).

## Conflict of interest

The authors declare that the research was conducted in the absence of any commercial or financial relationships that could be construed as a potential conflict of interest.

## Publisher's note

All claims expressed in this article are solely those of the authors and do not necessarily represent those of their affiliated organizations, or those of the publisher, the editors and the reviewers. Any product that may be evaluated in this article, or claim that may be made by its manufacturer, is not guaranteed or endorsed by the publisher.

## Abbreviations

FD: Flow diverter
OCT: Optical coherence tomography
DSA: Digital subtractive angiography
CM: Communicating malapposition
PCI: Percutaneous coronary intervention.
